# Work in Later Life: Structures and Spaces/Barriers and Benefits

**DOI:** 10.1093/geroni/igaf122.1816

**Published:** 2025-12-31

**Authors:** Anne Barrett, Dawn Carr

## Abstract

Despite the prevailing image of later life as centered on leisure, work – whether paid or unpaid – remains a core institution and activity in the lives of older adults. This symposium brings together multidisciplinary research conducted at both macro- and micro-levels to explore a range of contemporary issues surrounding work in later life, including the legal system’s role in protecting older workers, the impact of technology on paid and unpaid work, the influence of the built environment on work patterns, and the health effects of paid work in later life. Focusing on the macro-level, Nathan examines how the legal framework meant to protect older workers can sometimes reinforce inequities, a concern that is growing with the rise of artificial intelligence in the workplace. Also focusing on the macro-level, Barrett and colleagues analyze how U.S policy documents depict age tech as a tool for reducing work and delivering better care, despite some evidence suggesting the opposite. DeJohn and colleagues examine spatial patterns in work, finding that public transit investments benefit employed older adults, while inadvertently displacing retirees. Addressing work at the micro-level, Cao and Carr investigate the effect of occupational autonomy on cognitive health and find that its benefits are greatest among more educated older adults. Together, these studies illuminate how work, as both an institution and activity, structures older adults’ lives and impacts their quality of life.

